# Towards a threat assessment framework for apps collusion

**DOI:** 10.1007/s11235-017-0296-1

**Published:** 2017-03-07

**Authors:** Harsha Kumara Kalutarage, Hoang Nga Nguyen, Siraj Ahmed Shaikh

**Affiliations:** 1 The Centre for Secure Information Technologies, Queen’s University of Belfast, Belfast, UK; 2 Centre for Mobility and Transport Research, Coventry University, Coventry, CV1 5FB UK

**Keywords:** Android security, Apps collusion, Threat assessment, Bayesian, Statistical modelling

## Abstract

App collusion refers to two or more apps working together to achieve a malicious goal that they otherwise would not be able to achieve individually. The permissions based security model of Android does not address this threat as it is rather limited to mitigating risks of individual apps. This paper presents a technique for quantifying the collusion threat, essentially the first step towards assessing the collusion risk. The proposed method is useful in finding the collusion candidate of interest which is critical given the high volume of Android apps available. We present our empirical analysis using a classified corpus of over 29,000 Android apps provided by Intel Security^TM^.

## Introduction

The current PBSM for Android has a rather narrow focus on individual malicious apps, and as it stands has no means to control flow of information or activity that may occur across apps. App collusion is an emerging threat [1] which can be exploited by aggregating permissions, using covert or overt channels between apps to achieve a malicious goal [2].

Existing security solutions would fail to detect such attacks [3], and there is no evidence to suggest that new app security protection mechanisms^1^ by Google™would address collusion.

**Fig. 1:**
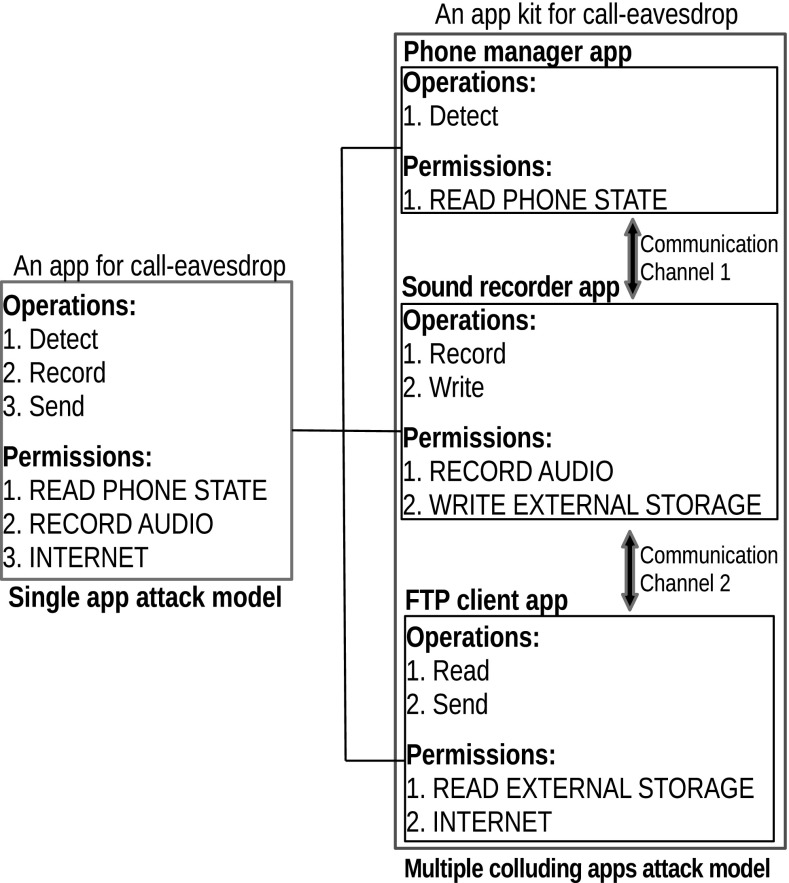
An example of permissions and operations being split between colluding apps for call-eavesdrop. Channel 1 can be an intent based communication while channel 2 can be a communication via external storage

This paper contributes towards a practical automated threat intelligence system for app collusion. The first contribution is a systematic threat assessment mechanism where we extend the current assumption of a single malicious app attack model to address a *set of multiple colluding apps* (see Fig. 1) and hence estimate the colluding threat using a concise definition. The second contribution is a computationally efficient filtering algorithm to filter out collusion candidates of interest using various possible threat assessment techniques.

### Rest of this paper

The rest of this paper is organised as follows. Section 2 provides an overview of the related work. Section 2.1 is a review of Android malware detection techniques and their suitability to employ in this particular problem. Inter apps communication is an integral part of collusion, hence Sect. 2.2 provides a review of inter apps communication and information leakage detection methods. Section 3 lays down the foundation of our threat assessment framework. Section 3.1 defines the notion of collusion while in Sect. 3.2 using permissions to denote threats to be materialised in collusion context. Section 4 formulates our research questions with a view of evaluating possible threat estimation approaches discussed in Sect. 4.1. Section 4.2 includes the filtering algorithm. Section 5 describes the dataset used for our experiments paying due attention to their relevance to wider global trends in Sect. 5.1. The experimental setup is described in Sect. 6 along with results. Section 7 reflects on the results and Sect. 8 concludes the paper.

## Related work

Android malware detection has been an attractive and active research area during last few years. As a result techniques for detecting Android malware are largely available [4, 5], but most of them target single malicious apps. The notion of collusion has recently been discussed in many research papers. A practical demonstration of collusion attacks through covert channels can be found in [2, 6]. Authors analyse free apps from the Android market and show that app collusion is a real threat. Soundcomber [7] is also a similar effort.

### Detecting malicious applications

In general, techniques for detecting Android malware are categorised into two groups: static and dynamic. In static analysis, certain features of an app are extracted and analysed using different approaches such as machine learning techniques. For example, Kirin [8] proposes a set of policies which allows matching permissions requested by an app as an indication for potentially malicious behaviour. DREBIN [9] trained Support Vector Machines for classifying malwares using number of features: used hardware components, requested permissions, critical and suspicious API calls and network addresses. Similar static techniques can be found in [10–14]. Conversely, dynamic analysis detects malware at the run-time. It deploys suitable monitors on Android systems and constantly looks for malicious behaviours imposed by software within the system. For example, [15] keeps track of the network traffic (DNS and HTTP requests in particular) in an Android system as input and then utilises Naive Bayes Classifier in order to detect malicious behaviours. Similarly, [16] collects information about the usage of network (data sent and received), memory and CPU and then uses multivariate time-series techniques to decide if an app admitted malicious behaviours. A different approach to translate Android apps into formal specifications and then employing existing model checking techniques to explore all possible runs of the apps in order to search for a matching malicious activity represented by formulas of some temporal logic can be found in [17, 18].

### Detecting malicious inter-app communication

Current research mostly focuses on detecting inter-app communication and information leakage. DidFail [19] is a analysis tool for Android apps that detects possible information flows between multiple apps. Each APK is fed into the APK transformer, a tool that annotates intent-related function calls with information that uniquely identifies individual cases where intents are used in the app, and then transformed APK is passed to two other tools: FlowDroid [20, 21] and Epicc [22]. The FlowDroid tool performs static taint tracking in Android apps. That analysis is field, flow and context sensitive with some object sensitivity. Epicc performs static analysis to map out inter-component communication within an Android app. Epicc [22] provides flow and context sensitive analysis for app communication, but it does not tackle each and every possible communication channels between apps’ components. The most similar work to DidFail is IccTA [23] which statically analyses app sets to detect flows of sensitive data. IccTA uses a single-phase approach that runs the full analysis monolithically, as opposed to DidFail’s composition two-phase analysis. Didfail authors acknowledge the fact that IccTA is more precise than the current version of DidFail because of its greater context sensitivity. This supports our claim in Sect. 4.1.1 - “context would be the key” for improving the precision. FUSE [24], a static information flow analysis tool for multi-apps, provides similar functions as didFail and IccTA in addition to visualising inter-component communication (ICC) maps. DroidSafe [25] is a static information flow analysis tool to report potential leaks of sensitive information in Android applications.

ComDroid [26] detects app communication vulnerabilities. Automatic detection of inter-app permission leakage is provided [27]. Authors address three kinds of such attacks: confused deputy, permission collusion and intent spoofing and use taint analysis to detect them. An empirical evaluation of the robustness of ICC through fuzz testing can be found in [28]. A study of network covert channels on Android is [29, 30]. Authors show that covert channels can be successfully implemented in Android for data leakage. A security framework for Android to protect against confused deputy and collusion attacks is proposed [31]. The master thesis [32] provides an analysis of covert channels on mobile devices. COVERT [33] is a tool for compositional analysing inter-app vulnerabilities. TaintDroid [34], an information-flow tracking system, provides a real time analysis by leveraging Android’s virtualized execution environment. DroidForce [35], build upon on FlowDroid, attempts to addresses app collusion problem with a dynamic enforcement mechanism backed by a flexible policy language. However static analysis encourages in collusion detection due the scalability and completeness issues [3]. Desired properties for a practical solution include, but not limited to: characterising the context associated with communication channels with fine granularity, minimising false alarms and ability to scalable for a large number of apps.

## Collusion threat intelligence

This section presents our main definition for collusion. We concern ourselves with the use of permissions for apps to execute threats. Permissions offer both, means to identify which combinations of apps can potentially execute a threat, as in Sect. 3.1, and means to indicate the nature of the threat likely to materalise given the type of permission as in Sect. 3.2.

### Formal definition

In this paper, the notion of collusion informally refers to the ability for a set of apps to carry out a threat in a collaboration fashion. In existing works [2, 20, 23, 24, 24, 26, 36], collusion is usually associated with inter-app communications and information leakage. However, to the best of our knowledge, there is no evidence suggesting the difference between threats caused by single apps and colluding apps. Therefore, we consider that colluding apps can carry out any threat such as the known ones posed by single apps. This allows collusion to cover a broader set of threats applicable, especially, for mobile devices.

A threat is a set of actions that must be executed in a certain order. In this paper, therefore, they are modelled by partially ordered sets $(T,\le )$ where *T* is a set of actions and $\le$ specifies the order in which actions must be executed. When $(T,\le )$ is carried out, actions from *T* must be sequentially executed according to some total order $\le '$ (i.e., $\forall t_1,t_2 \in T: t_1 \le ' t_2 \vee t_2 \le ' t_1$) such that $\le \subseteq \le '$; in other words, $(T,\le ')$ is a total extension of $(T,\le )$. Let $Ex((T,\le ))$ denote the set of all possible total extensions of $(T,\le )$; i.e., all possible ways of carrying out the threat $(T,\le )$. We have $Ex((T,\le )) = \{ (T,\le ') \mid \,\le \subseteq \le ' \wedge \le ' \text { is total} \}$. To this end, a sequence of actions can be seen interchangeably as a totally order set. Furthermore, one may obfuscated a total extension of a threat by scattering it with meaningless or unrelated actions. However, the total extension must be a subsequence^2^ of the execution. Similarly, we also define an inter-app communication as a poset.

We define the notion of collusion based on the following axioms:

1. Actions are operations provided by Android API (such as record audio, access file, write file, send data, etc.). Let *Act* denote the set of all actions.
2. Actions can be characterised by a number of static or dynamic attributes such as permissions, input parameters, etc. For the purpose of this paper, we only consider permissions. Let *B* denote the set of all action attributes and $\textit{pms}:Act \rightarrow \wp (B)$ specify the set of permissions required to execute an action.
3. A threat $t = (T,\le )$ is a poset. Let $\tau$ denote the set of all threats. In the scope of this paper, $\tau$ represents the set of all known threats caused by single apps.
4. An inter-app communication $c = (C,\le )$ is a poset. Let *com* denote the set of all known inter-app communications.

#### Definition 1

A non-singleton set *S* of apps is colluding if they execute a sequence $A \in Act^*$ such that:

- $(d_1)$: there exists a subsequence $A'$ of *A* such that $A' \in Ex(t)$ for some $t \in \tau$; furthermore, $A'$ is collectively executed by every app in *S*, i.e., each app in *S* executes at least one action in $A'$; and
- $(d_2)$: there exists a subsequence $C'$ of *A* such that $C' \in Ex(c)$ for some $c \in com$.

### Threat quantification

As per our collusion definition in Sect. 3.1, estimating the collusion threat likelihood $L_{c}(S)$ of a non-singleton set *S* of apps involves two likelihood components $L_{\tau }(S)$ and $L_{com}$(S), where $L_{\tau }(S)$ denotes the likelihood of carrying out a threat in $\tau$ by apps in *S* and $L_{com}(S)$ denotes the likelihood of performing some inter-app communication in *com* between apps in *S*. Using the multiplication rule of well-known basic principles of counting:

1 $$\begin{aligned} L_{c}(S)= L_{\tau }(S) \times L_{com}(S) \end{aligned}$$

We apply some basic machine learning techniques in Sect. 4.1 to demonstrate the evaluation of Eq. 1.

## Research questions

This section lists some important collusion related research questions.

1. Is “Permission” a relevant attribute to use in threat quantification? The current security model of Android depends on permissions. Hence it would be naturally the first selection of features in any Android security discussion. We investigate if permissions is a relevant feature to use in our threat quantification model.
2. Which permissions inform in the threat model? If above RQ1 is true, we need to investigate which permissions would be far more informative in collusion threat estimation than others. We use a simple graphical technique for variable subset selection.
3. Can critical permissions be considered as more informative in threat estimation than non-critical permissions? Requesting a more critical permission increases likelihood of being malicious than requesting a less critical permission is a typical belief within the community. We will test this hypothesis using real data.
4. What techniques/methods can be applied to estimate the parameters of proposed threat quantification formula? Here we investigate possible deterministic and stochastic techniques.
5. Is there a correlation between different measures and collusion threat? We study the correlation between number of permissions, types of apps and collusion threat.
6. What percentage of app sets have collusion potential? In order to materialise a collusion, an app set has to satisfy desideratum $d_1$ and $d_2$ in Eq. 1. We investigate what percentage of app pairs satisfy $d_1$ and $d_2$.
7. What is the most likely threat to materialise in collusion context?

### Methods

This section discusses some possible methods in estimating $L_{\tau }$ and $L_{com}$ in order to evaluate Eq. 1.

#### Estimating $L_{\tau }$

Three possible approaches is proposed in estimating $L_{\tau }$: policy based, data driven and modelling. Each approach has inherent pros and cons. The sole purpose of presenting three different approaches in this work is to explore the reader the ability to employ them in estimating $L_{\tau }$ under different situations and constraints. An evaluation of which approach is superior to others is out of the scope of this paper. Such an evaluation depends on number of factors such as domain knowledge, data availability, accuracy requirements and computational cost.

*Policy based:*

A set of rules is defined utilising the knowledge about aforementioned attributes in axiom $A_2$. We use Kirin [8] rule set ${\mathcal {K}}$ for the empirical analysis presented in this paper. Each security rule $r\in {\mathcal {K}}$ is defined using permissions to enforce a stated security policy. The following check was performed in estimating $L_{\tau }(S)$:

$$\begin{aligned} \theta _r(S) \equiv \bigwedge _{S' \subset S} r \not \subseteq \bigcup _{a \in S'} \textit{pms}(a) \wedge r \subseteq \bigcup _{a \in S} \textit{pms}(a) \end{aligned}$$

Note that $\theta _r(S)$ is equal to *unity of ability* 3 to pose a predefined threat for single apps by the app set *S*, as any matching rule is an indication of a malicious effect regardless of the threat type. The ability to bypass a single rule $\Leftrightarrow$ ability to pose a predefined threat by the app set S. Assuming that number of rules defined for threat definitions in the database is exclusive and exhaustive,

2 $$\begin{aligned} L_{\tau }(S)=\frac{\sum _{r \in {\mathcal {K}}} \theta _r(S)}{|{\mathcal {K}}|} \end{aligned}$$

Inherent weakness associated with this approach is its inability to capture the *motivation* uncertainty behind an operation. For example, SEND_SMS can be used maliciously as well as legitimately needed by communication apps. The problem here is how to capture this kind of uncertainty by a rule defined based on predefined policies? In fact context would be the key for capturing the motivation, and extra ordinary security can be achieved only through listening to all information sources (including contextual parameters) on the device. However defining finite number of white (or black) list rules using large number of attributes to describe each and every possible state of the device with respect to the context is not feasible. Defining rules requires expertise knowledge as well as tedious human involvements, and on the other hand context is dynamically evolving. Possibility to cover future threats imagined by experts, but not yet executed by any attacker, would be a strength of this approach.

*Data driven:*

In many classification problems, explicit rules do not exist but examples can be obtained easily. Hence a classifier cannot be constructed from known rules and therefore one tries to infer a classifier from a (limited) set of training examples. The use of examples thus elevates the need to explicitly state the rules for the classification by the user [37]. Here we use Bayesian fusion - well known log likelihood model^4^ for this purpose. Bayesian fusion has been widely used in intrusion detection [39, 40]. The aim is to use only data, instead of defining rules, in computing $L_{\tau }(S)$ while capturing motivation uncertainty. Let *H* be the hypothesis that *S* satisfies the condition $d_1$ of definition 3.1 and assume mutually independent attributes in *B*. Then *H* can be tested using log-likelihood model as follows.

3 $$\begin{aligned} ln\frac{P(H/B_S)}{P(\lnot H/B_S)}=ln\frac{P(H)}{P(\lnot H)}+\sum _{k \in B_S}ln\frac{P(b_k/H)}{P(b_k/\lnot H)} \end{aligned}$$

where $B_S$ denotes the set of permissions required by apps in *S*, i.e., $B_S = \bigcup _{a \in S} \textit{pms}(A)$. Then, $L_{\tau }$ is defined as,

4 $$\begin{aligned} L_{\tau }(S)={\left\{ \begin{array}{ll} ln\frac{P(H/B_S)}{P(\lnot H/B_S)} &{} \text { if } ln\frac{P(H/B_S)}{P(\lnot H/B_S)}>0 \\ 0&{} \text { otherwise} \end{array}\right. } \end{aligned}$$

Here, the term $P(H/B_S)$ explains how likely the app set *S* satisfies required operations for producing threat in $\tau$ given feature set $B_S$. $P(\lnot H/B_S)$ denotes the negation of $P(H/B_S)$. Note that even if our attack model is multiple apps, $d_1$ focuses only on operations required to execute a threat (e.g. Detect, Record and Send in Fig. 1) by a single app attack model. Hence it is possible to use known clean and malicious single apps to train the classifier in estimating $L_{\tau }$ as large collections of colluding apps found in the wild are not available at present for training and testing purposes. Obviously some additional operations (e.g. Read and Write in Fig. 1) may require to execute the same threat in “multiple colluding apps model”. But such operations are connected to the inter-app communication element in our threat model, and hence covered by $L_{com}$ and not affected on $L_{\tau }$.

Since it learns both benign and malicious nature of attributes in *B* from existing data (training data), no expert’s effort needed for defining rules, and can simply adopt number of different features in *B* (e.g. category, developer information and many other static attributes) to inform benign and malicious nature even in different contexts. This likelihood estimating strategy has two advantages. First, the malicious nature in multiple attributes are combined not in an ad-hoc, but rather in a data-driven manner. Secondly, it allows raising alarms on malicious behaviours that are not by themselves appearing to be malicious in any single attribute. However any threat not in the training data will not be covered unless explicitly incorporated them with the model.

*Modelling approach:*

In order to estimate $L_{\tau }$, we employ a so-called Naive Bayesian informative [41] model. Naive Bayesian informative is extensively used for modelling the knowledge which is not available in data (e.g. semantic information such as permissions’ critical level). To this end, we consider a multi-variate random variable $Y = (y_1, \dots , y_k)$. Here, *k* is the total number of permissions in Android OS and $y_j \in \{0,1\}$ are independent Bernoulli random variables. A variable $y_j$ takes the value 1 if permission *j* is found in *S*, 0 otherwise. With this, *P*(*Y*) stands for the probability of obtaining *S* with permissions as described by *Y*. Our probabilistic model is then given by Eq. (5):

5 $$\begin{aligned} P(Y)=\prod _{j=1}^{k}\lambda _{j}^{y_{j}}(1-\lambda _{j})^{1-y_{j}} \end{aligned}$$

where $\lambda _j \in [0,1]$ is the Bernoulli parameter.

In order to compute $L_{\tau }$ for a given set *S*, we average out the function $ln \{(P(Y))^{-1}\}$ using number of permissions in set *S* and scale down it to the range [0,1] for comparisons. The desired goal is to make requesting a more critical permission to increase likelihood of “being malicious” than requesting a less critical one even though the two permissions have similar frequencies. Therefore, any monotonically decreasing function can be chosen [41].

To complete our modelling, we need to estimate values $\hat{\lambda _{j}}$ that replace $\lambda _{j}$ in the computation of $L_\tau .$ To this end – to avoid over fitting *P*(*Y*) – we estimate $\lambda _{j}$ using informative beta prior distributions [42] and define the maximum posterior estimation

6 $$\begin{aligned} \hat{\lambda _{j}}=\frac{\sum y_{j}+ \alpha _{j}}{N+ \alpha _{j}+\beta _{j}} \end{aligned}$$

where *N* is the number of apps in the training data set and $\alpha _j, \beta _j$ are the penalty effects. In this work we set $\alpha _j =1.$ The values for $\beta _j$ depend on the critical level of permissions as given in [41, 43]. $\beta _j$ can take either the value 2*N* (most critical), *N* (critical) or 1 (non-critical).

#### Estimating $L_{com}$

In order to materialise a collusion, desideratum $d_2$ definition 1 should also be satisfied - there should be an inter app communication *closely related* to the threat mentioned in $d_1$. To establish this association we need to consider number of factors including the contextual parameters. At this stage of the research we do not focus on estimating the strength of connection (association) between $d_2$ and $d_1$. In this work we investigate what percentage of communication channels can be detected through static code analysis, and simply assume these channels can be used for malicious purpose by apps in set *S*. Hence we consider $L_{com}$ to be a binary function such that $L_{com} \in \{1,0\}$ which takes the value 1 if there is inter app communication within *S* using either intents or external storage (we do not investigate other channels in this work).

### Filtering for collusion candidates

The search space posed by possible app combinations is very large. Therefore it is not computationally cheap doing deep analysis on each and every app pairs. Effective methods are needed to narrow down the search space to collusion candidates of interest.

**Fig. 2:**
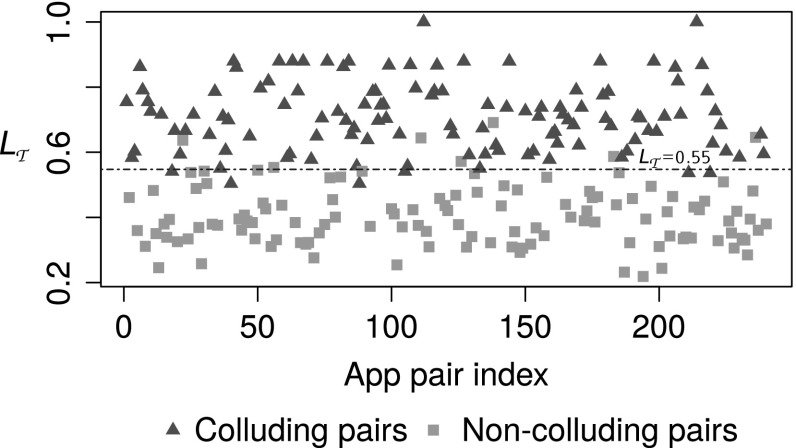
Validation: threat score obtained by each pair in the validation data set

Our filtering mechanism consists of two sub filters: inner and outer. Inner filter applies on top of the outer filter. Outer filter is based on $L_{\tau }$ value which can be computed using permissions only. Permissions are very easy and cheap to extract from APKs - no decompilation, reverse engineering, complex code or data flow analysis is required. Hence outer filter is computationally efficient. Majority of non-colluding app pairs in an average app set can be pruned out using this filter (see Fig. 2). Hence it avoids doing expensive static/dynamics analysis on these pairs. Inner filter is based on $L_{com}$ value which should be computed using static code analysis. A third party research prototype tool Didfail [19] was employed in finding intent based inter app communications. A set of permission based rules was defined to find communication using external storage. Algorithm 1 presents the proposed app filtering mechanism for colluding candidates of interests.
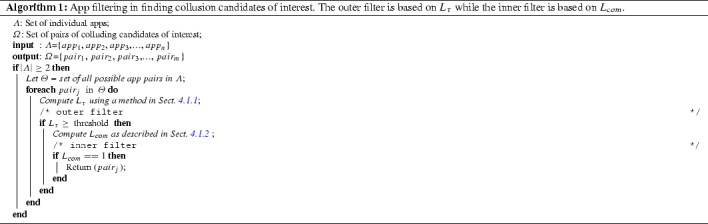


## Dataset description

The malicious app set uses in this paper is significantly a larger than many studies in the literature [44]. In this work we perform our analysis using a 29k+ size app set which includes “malicious”, “potentially malicious” and “ clean” apps carefully classified experts in Intel Security^5^. That sample prduces 420+ millions app pairs for pairwise analysis in this work. With a huge sample it is possible to know computed statistics with a lot of precision even the data is very scattered, and hence more accurate inferences about the population. Table 1 presents the descriptive statistics which tells us each app category has different permission distribution.

### Dataset vs global trend

Table 2 presents a comparison of top 10 most used permissions between each app category as a percent of apps that requested those permissions and rank within the group. These statistics are similar to the other works in the literature which have been used different data sets for computing the same (e.g. [43]).

**Table 1:** Descriptive statistics - “number of permissions” is the variable

	Potentially	Clean	Malicious	Whole
Num.apps	9976	9476	9770	29222
Minimum	0	0	0	0
Maximum	76	94	99	99
Median	11	10	10	10
Mean	11.76	12.77	12.38	12.30
Variance	38.39	139.05	84.04	86.46

**Table 2:** Top 10 most used permissions in each app category

Permission name	Malicious (Rank)	Potentially (Rank)	Clean (Rank)
INTERNET	98 (1)	100 (1)	82 (1)
ACCESS_NETWORK_STATE	95 (2)	98 (2)	76 (2)
WRITE_EXTERNAL_STORAGE	75 (4)	69 (5)	66 (3)
WAKE_LOCK	49 (6)	47 (9)	55 (4)
READ_PHONE_STATE	85 (3)	93 (3)	48 (5)
ACCESS_WIFI_STATE	70 (5)	73 (4)	43 (6)
GET_ACCOUNTS	29 (12)	40 (11)	43 (7)
VIBRATE	38 (10)	44 (10)	40 (8)
RECEIVE_BOOT_COMPLETED	41 (9)	62 (7)	33 (9)
ACCESS_FINE_LOCATION	42 (8)	55 (8)	28 (10)
ACCESS_COARSE_LOCATION	48 (7)	62 (6)	26 (11)

Nine permissions occurred in all three top 10 lists in common. A total of 11 permissions are included. Percentages and rank within the group is presented

## Experimental setup and results

Algorithm 1 was automated using R^6^ and Bash scripts. It also includes calls to a third party research prototype [19] to find intent based communications in computing $L_{com}$. A set of permission based security rules was defined to find communication using external storage. The likelihood ($P(b_k/H)$, $P(b_k/\lnot H)$) and prior (*P*(*H*), $P(\lnot H)$) distributions in Eq. 3 were estimated using the “clean” and “malicious” app sets. Model parameter in Eq. 5 was also estimated using the same data set. Average processing time per app pair was recorded as 80s - outer filter ($\le 1s$) and inner filter (79s). Average time was calculated on a mobile workstation with an Intel Core i7-4810MQ 2.8GHz CPU and 32GB of RAM.

**Table 3:** Confusion matrix for log likelihood method

n=240	Actual colluding	Actual non-colluding
Predicted colluding	92	44
Predicted non-colluding	28	76

### Validation

Our validation data set consists of 240 app pairs in which half (120) of them are known colluding pairs while the other half non-colluding pairs. In order to prevent over fitting, app pairs in the validation and testing sets were not included in the training set. Table 3 presents the confusion matrix for the log-likelihood method. Different performance measures such as sensitivity = 0.77, specificity = 0.63, precision = 0.68 and F-score^7^ = 0.72 were computed for log-likelihood method. As shown in Fig. 2 proposed naive Bayesian method assigns higher threat scores (in fact $L_{\tau }$, assuming communication, i.e. $L_{com}=1$, for each pair) for colluding pairs than clean pairs. Table 4 presents the confusion matrix obtained for the naive Bayesian method by fitting a linear discriminant line (blue dotted) in Fig. 2. Sensitivity = 0.95, specificity = 0.94, precision = 0.94 and the F-score = 0.95 for the naive Bayesian method. Error rates obtained by this method against the validation dataset are encouraging, 3% false positives and 2.5% false negatives. These error rates are a big improvement but still too high for practical use. However policy based method detected only two colluding pairs (true positives) in the validation set. This may be due to the limitations of the rule set which is not exhaustive.

**Table 4:** Confusion matrix for naive Bayesian method

n=240	Actual colluding	Actual non-colluding
Predicted colluding	114	7
Predicted non-colluding	6	113

**Table 5:** Testing the proposed filter

ID	1	2	3	4	5	6	7	8	9	10	11	12	13	14
1		**0.51**	*1.61*	*0.97*	*1*	*0.8*	*1*	***0.81***	*0.77*	*0.77*	*0.77*	*0.44*	*0.44*	*0.95*
2			*0.48*	*0.62*	*0.55*	*0.49*	*0.55*	***0.58***	***0.51***	***0.51***	*0.58*	*0.31*	*0.31*	*0.49*
3				0.69	0.64	0.56	*0.64*	*0.48*	*0.61*	*0.61*	*0.72*	*0.41*	*0.41*	*0.58*
4					*1*	*0.84*	*1*	*0.85*	*0.71*	*0.71*	*0.82*	*0.56*	*0.56*	*0.95*
5						*0.84*	*1*	*0.86*	*0.67*	*0.67*	*0.82*	*0.47*	*0.47*	*1*
6							*0.84*	*0.68*	*0.58*	*0.58*	*0.65*	*0.43*	*0.43*	*0.78*
7								0.86	**0.67**	*0.67*	*0.82*	*0.47*	*0.47*	*1*
8									0.51	***0.51***	*0.58*	*0.31*	*0.31*	*0.77*
9										*0.77*	*0.77*	*0.44*	*0.44*	*0.61*
10											*0.77*	*0.44*	*0.44*	*0.61*
11												*0.47*	*0.47*	*0.73*
12													**0.47**	*0.41*
13														*0.41*
14														

For readability – we leave the lower half empty since the table is symmetric. Underlined values shows true positives, Bolditalic values shows false positives, Italic values shows true negatives, and Bold values shows false negatives

### Testing

We tested our filtering mechanism with a different sample consists of 91 app pairs. Table 5 presents the outcome. Each cell denotes the $L_{\tau }$ value for the corresponding pair. To minimise false negatives, we use the lower bound (=0.50) gained from the validation data set for the discriminant line as threshold for $L_{\tau }$. We report possible collusion if $L_{\tau }\ge 0.5$ and $L_{com}=1$, otherwise we report non-collusion. This yields symmetric data – for readability we leave the lower half of the matrix empty. Underlined values shows true positives, Bolditalic values shows false positives, Italic values shows true negatives, and Bold values shows false negatives.

With regards to false alarms, app pair (1,2) was not detected by our analysis due to the third party tool does not detect communication using SharedPreferences. Since we do only pairwise analysis, app pair (7,9) was not reported. That pair depends on transitive communication. Pair (12,13) was not reported since $L_{\tau }$ is less than the chosen threshold. It is possible to reduce false alarms by changing the threshold. For example either setting the best possible discriminat line or its upper bound (or even higher, see Fig. 2) as the threshold will produce zero false positves or vice versa. But as a result it will increase false negative rate that will affect on the F-score - the performance meassure of the classifier. Hence it would be a trade-off between a class accuracy and overall performance. However since the base rate of colluding apps in the wild is close to zero as far as anyone knows, the false positive rate of this method would have to be vanishingly small to be useful.

Precise estimation of $L_{com}$ would be useful to reduce false alarms in our analysis. But it should be noted that existence of a communication is only a necessary condition to happen a collusion, but not a sufficient condition to detect it. In this context it is worth to mention that a recent study [45] shows that 84.4% of non-colluding apps in the market place can communicate with other apps either using explicit (11.3%) or implicit (73.1%) intent calls. Therefore the threat element (i.e. $L_{\tau }$) is far more informative in collusion estimation than the communication element ($L_{com}$) in our model.

Both validation and testing samples are blind samples and we have not properly investigated them for the biasednes or realisticity.

**Table 6:** Most requested permissions: the top 10 dissimilarity scores

Permission name	Malicious (Rank)	Potentially (Rank)	Most Critical
READ_PHONE_STATE	36.85 (1)	44.82 (1)	N
ACCESS_WIFI_STATE	26.97 (2)	30.25 (3)	N
ACCESS_COARSE_LOCATION	22.34 (3)	36.43 (2)	Y
ACCESS_NETWORK_STATE	19.32 (4)	21.96 (8)	N
INTERNET	15.82 (5)	17.59 (9)	N
READ_HISTORY_BOOKMARKS	14.03 (6)	22.65 (7)	N
ACCESS_FINE_LOCATION	13.96 (7)	26.65 (5)	Y
SYSTEM_ALERT_WINDOW	13.72 (8)	12.36 (13)	N
MOUNT_UNMOUNT_FILESYSTEMS	11.53 (9)	5.70 (16)	N
GET_TASKS	11.23 (10)	8.49 (15)	N

**Table 7:** Least requested permissions: the bottom 10 dissimilarity scores

Permission name	Malicious (Rank)	Potentially (Rank)	Critical
MANAGE_ACCOUNTS	−13.76 (1)	−15.71 (1)	N
GET_ACCOUNTS	−13.75 (2)	−2.58 (26)	Y
USE_CREDENTIALS	−13.59 (3)	−15.22 (2)	N
READ_SYNC_SETTINGS	−11.65 (4)	−12.66 (4)	N
READ_CONTACTS	−10.96 (5)	−13.71 (3)	Y
WRITE_SYNC_SETTINGS	−10.37 (6)	−11.50 (5)	N
AUTHENTICATE_ACCOUNTS	−8.97 (7)	−10.01 (7)	N
NFC	−6.06 (8)	−6.30 (12)	Y
WAKE_LOCK	−5.93 (9)	−7.77 (9)	N
BIND_REMOTEVIEWS	−5.77 (10)	−5.89 (13)	N

**Fig. 3:**
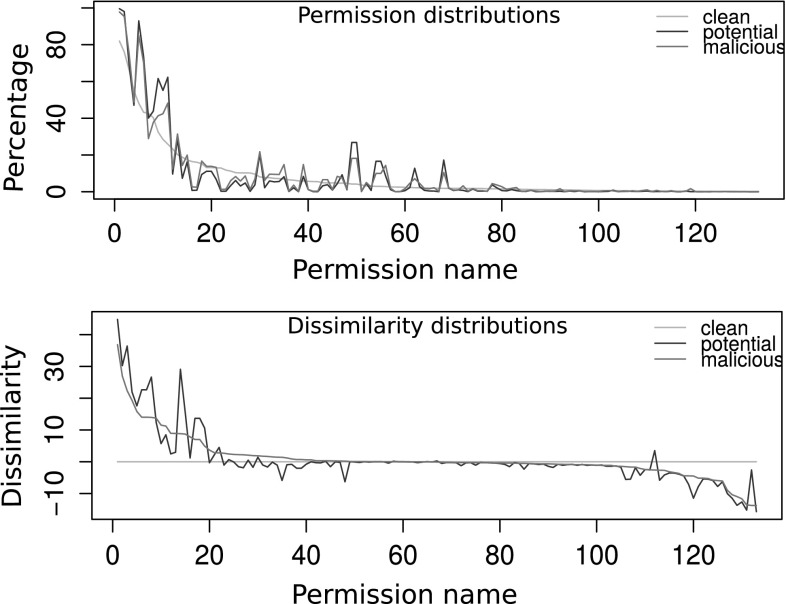
Permission distributions: variable “permission” has three different distributions in each category (*top*). Dissimilarity scores for malicious and potentially malicious classes against the clean set (*bottom*)

### RQ1. Is “Permission” a relevant attribute to use in threat quantification?

As shown in Fig. 3, each app category has different distributions over all permissions in Android. Therefore permissions can be used as an indicator to classify malicious and benign nature of apps, and hence as an element in *B*. The bottom graph in Fig. 3 presents the dissimilarity scores - the amount of deviations of distributions of malicious and potentially malicious groups from the clean set.

### RQ2. Which permissions inform in the threat model?

As in left and right tails of the bottom graph of Fig. 3, there are some certain group of permissions which malicious apps are *most* and *least* likely requested than clean apps. Tables 6 and 7 list names of top 10 such permissions from each group respectively. Only those (i.e. most and least likely) permissions help in threat estimation or app classification as other permissions are used in a similar manner by both malicious and clean apps.

### RQ3. Can critical permissions be considered as more informative in threat estimation than non-critical permissions?

Requesting a more critical permission increases likelihood of being malicious than requesting a less critical permission is a typical assumption in many permission based security solutions (e.g. [41, 43]). In [43], requesting a critical permission is viewed as a signal that the app is risky. Tables 6 and 7 compare top 10 most and least likely requested permissions with critical permissions listed in [41, 43]. Only two overlaps are in the most requested list (see third column of Table 6). Three least requested permissions by malicious apps have been included in the critical permission list as well (see third column in table 7). So, it is essential to incorporate semantic information in classification model to classify apps using critical permissions. Otherwise they might not be a useful feature in classifying apps between malicious and benign as they are equally requested by both categories. Instead, most and least likely requested permissions would inform more in the classification model.

### RQ4. What techniques/methods can be applied to estimate the parameters of proposed threat quantification formula?

This question was answered in Sects. 4.1,  6.1 and 6.2. Three different methods proposed, validated and tested.

### RQ5. Is there a correlation between different measures and collusion threat?

#### Number of permissions vs threat

**Fig. 4:**
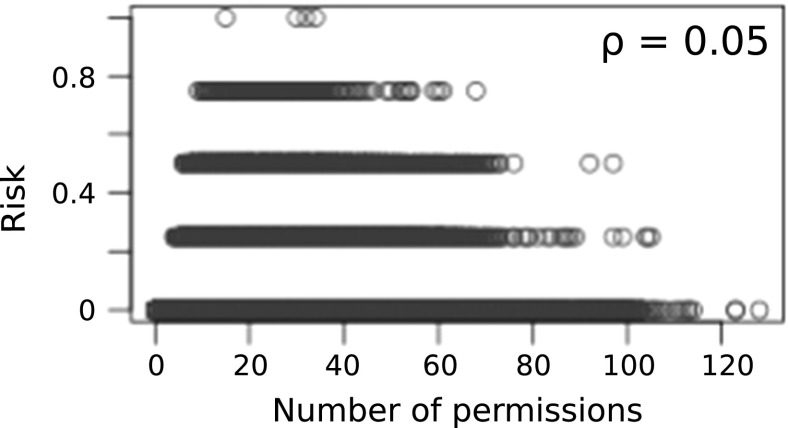
Correlation: number of permissions vs threat estimated using policy based model

**Fig. 5:**
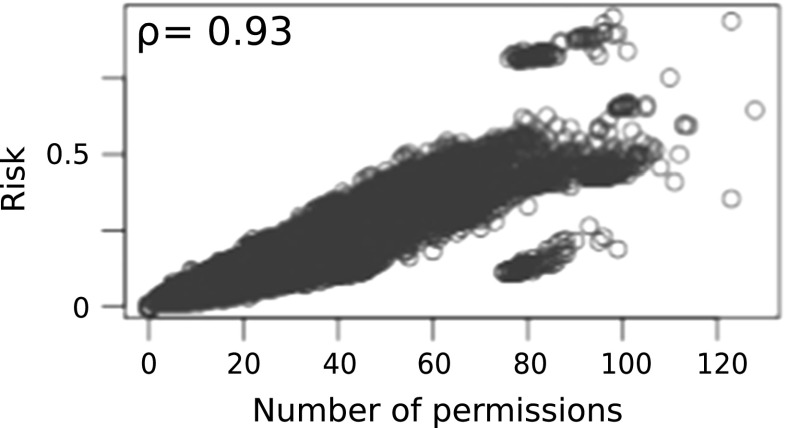
Correlation: number of permissions vs threat estimated using naive Bayesian model

**Fig. 6:**
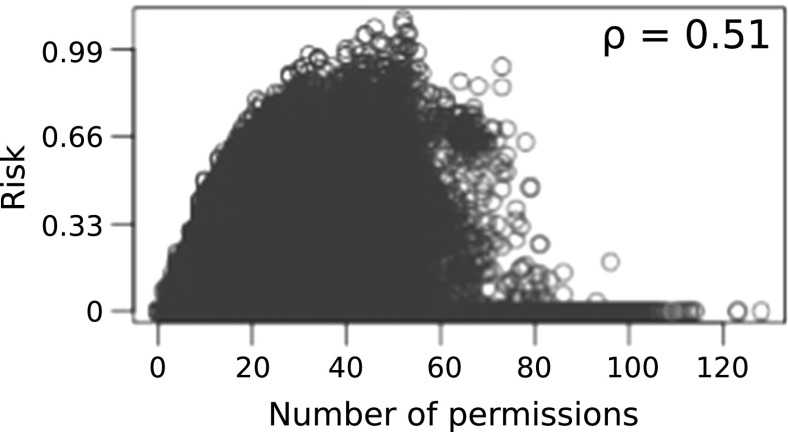
Correlation: number of permissions vs threat estimated using log likelihood model

Correlation between number of permissions in *S* and $L_{c}$ is investigated. The idea is to investigate the feasibility of using number of permissions as a risk signal for the collusion threat. Figures 4, 4 and 6 show plots of threat scores of each approach. $\rho$ denotes the Pearson correlation coefficients. As shown in Fig. 5 threat estimation using naive Bayesian model exhibits a higher correlation with number of permissions in *S* than other two methods. There is no correlation in policy based method. This might be due to the rule set is not exhaustive. A weak correlation can be found in log likelihood method. However it should be noted that a strong correlation does not mean the goodness of fit of the model for the purpose.

#### Type of apps vs threat

Table 8 presents the distribution of risky pairs against the type of individual apps in a pair. Three types of classification is considered as shown in table 8. As per the table 8, including a malicious or a potentially malicious app in a pair increases the collusion potentially. However since apps detected as malicious are quickly removed from app stores, collusion in practice may not manifest as such in the real world.

**Table 8:** Distribution of percentage (%) of risky pairs over each risk estimation methods and app type

App pair consists of	Policy based	Naive Bayesian	Log likelihood
Two clean apps	11	9	24
At least one potentially app	33	33	36
At least one malicious app	56	58	40
At least one potentially or malicious app	78	77	54

### RQ6. What percentage of app sets have collusion potential?

#### Possible channels

What are possible channels for satisfying $d_2$, and what percentage of them can be detected through static code (including permissions) analysis is investigated. Possible channels to communicate between two apps would be:

- Intents (static code analysis needed)
- External storage (permission analysis sufficient), only READ and WRITE permissions are needed
- Content providers (static code analysis needed)
- Shared Preferences (static code analysis needed)
- Sockets (static code analysis needed)

As we found in this analysis 45.7% of app pairs can communicate through external storage, and 3.2% of app pairs (within the clean set) can communicate through explicit intents. These figures are not surprising as recent study [3] shows that 84.4% of clean apps in the market place can communicate with other third party apps either using explicit (11.3%) or implicit (73.1%) external intent calls.

**Table 9:** Top 5 matching rules

Rule	%
ACCESS_COARSE_LOCATION, INTERNET, RECEIVE_BOOT_COMPLETED	32.01
ACCESS_FINE_LOCATION, INTERNET, RECEIVE_BOOT_COMPLETED	30.81
INSTALL_SHORTCUT, UNINSTALL_SHORTCUT	13.94
READ_PHONE_STATE, RECORD_AUDIO, INTERNET	13.28
SEND_SMS, WRITE_SMS	5.00

#### Collusion potentially

Policy based model classified as 7% of app pairs has collusion potentially. Log likelihood ratio classified as 16% of app pairs has that ability. Naive Bayesian assigned a threat score greater than 0.8 for 20% of app pairs. These figures may include some false positives as we don’t estimate $L_{com}$ preciously as mentioned above.

### RQ7. What is the most likely threat to materialise in collusion context?

A rule in the policy based model describes a possible threat. Hence it is possible to compute the most likely threat to be materialised in the collusion context by counting the number of app pairs matched against each rule. Table 9 presents the top 5 matching rules. As it is obvious, 76% of matching accounts for 1st, 2nd and 4th rules in the table. The main purpose of those three rules to prevent information leakage. Hence the most likely threat to be materialised through collusion would be information leakage.

## Discussion

There is a gap for a better risk communication model in the current PBSM of Android [41, 43]. It presents the risk of “to be installed apps” in the form of “dangerous permissions combinations”, but underestimates the associated risk of app collusion. We argue any future model needs to take into account possible app collusion and should communicate the risk in a way users can easily understand and compare with other competitive apps providing similar functions. In this work we quantify the threat using Eq. 1, by taking into account possible app collusion, and present that threat in *numerical* forms. We believe that only then users can compare, limit and ultimately better manage the risks associated with installing un-trusted apps.

The evaluation of the proposed threat quantification method depends on a mix of speculative reasoning and an empirical analysis. This is mainly due to lack of large number of known colluding app samples are available for training and testing purposes. This is a major constraint for advances in this research topic. Dividing the likelihood $L_c$ in Eq. 1 into two parts, i.e. $L_{\tau }$ and $L_{com}$, helps to overcome this issue. Loosely speaking, most threats are common in single and multiple apps attack models, and some additional operations are required to establish communication channels to execute the same threat in multiple apps attack model (see Fig. 1). Operations required to establish communication channels are covered by $L_{com}$ in our threat quantification model, while rest of operations are covered by $L_{\tau }$. Hence $L_{\tau }$ can be trained using existing datasets for single apps. However there may be threats applicable only for collusion scenarios and cannot execute under single app attack model. Such cases may need to identify and explicitly train in the models.

Overwhelming number of possible app pairs available in an app market presents a huge challenge to a fully automated collusion detection system; collusion across three or more apps makes this problem worse. Therefore computationally efficient methods are required to reduce the size of target sets for details analysis. Proposed method useful herein reducing search space as it looking for interesting collusion candidates, eliminating apps that are unlikely to be malicious, and focusing on those with a higher probability. For example, it reduces the sample size of 29k apps by 93% using the policy based method and 84% using log likelihood method (see Sect. 6.8.2). Given a overwhelming number of possible app pairs in an app market such a reduction is very welcome.

Threat probability calculation using Naive Bayesian model reduces false alarms substantially. For example, against the set of 240 colluding and non-colluding app pairs, it reported 3% false positives and 2.5% false negatives (see Table 4). Though these error rates are a big improvement, still may be a too high for a practical usage given that huge number of app pairs in an app market. Further reduction is needed.

Since the base rate of colluding apps in the wild is close to zero as far as anyone knows, the outer filter (see algorithm 1) itself can filter out most of innocent app pairs form a large app set for a minimal computational cost (processing time per app pair $\le 1s$). Hence proposed filter is relatively an efficient.

## Conclusion

App collusion is possible because the current security mechanism on Android is not focused on controlling inter-app communications (IACs). Instead it has been designed based on intra and inter app communications. IAC plays a vital role in enabling legitimate functions for an app. However the same can be used for malicious purposes as well. The technical challenges associated with any proposal for collusion detection is to tackle this *uncertainty*. Inability to solve this problem may result in a high number of false alarm rates. For example, as shown in [3], XManDroid [46] has a very high false positive rate (55%) which defines classification policies based on certain permissions combinations. As shown in our work, employing probabilistic techniques provides a promise, but further reduction is needed in false alarms. Our hypothesis - taking some countermeasures such as contextual anomaly detection (more attributes in *B*) and estimating $L_{com}$ preciously will reduce false alarms further. Some possible attributes for this task would be type of data on the channel (e.g. image, binary, text), payload size and type of the channel (e.g. HTTP GET requests vs. POST or content provider reads vs. writes), developer information (e.g. same developer, high-visibility developers such as Google and Facebook), app category (e.g. game, weather) and presence of encryption. An extensive study is needed this regard and left as a future work.

Finally, app collusion on the PBSM is a consequence of the basic assumption on which the permission based model relies that apps can be independently restricted in accessing resources and then safely composed on a single platform. As discussed in this paper this assumption is incorrect and app collusion can be exploited to break the permission based model. Therefore permissions should be granted and managed under the assumption that apps can aggregate their permissions by colluding over communication channels. Any future model needs to take into account possible app collusion and should communicate the risk in a way users can easily understand and compare with other competitive apps providing similar functions. Proposed threat quantification mechanism in this paper provides a promise towards this direction.

## Footnotes

1 With “verify apps” turned on, apps will be scanned at the time of installation as well as periodically thereafter individually for potentially harmful behaviour.

2 A sequence $a_1\ldots a_n$ is a subsequence of another one $b_1\ldots b_m$ iff $a_1\ldots a_n$ can be obtained from $b_1\ldots b_m$ by deleting some elements $b_i$’s.

3 As each rule in the set defined to protect the device/user from a specific predefined threat

4 Neyman Pearson lemma demonstrates that such a test has the highest power among all competitors when parameters are known [38].

5
http://www.mcafee.com/us/index.html.

6
http://www.r-project.org/.

7 A measure of accuracy of a method.
